# Pyloric atresia: a challenge in an underdeveloped country

**DOI:** 10.11604/pamj.2017.28.210.14102

**Published:** 2017-11-07

**Authors:** Aloise Sagna, Ndeye Aby Ndoye, Cheikh Diouf, Papa Alassane Mbaye, Mbaye Fall, Azhar Salim Mohamed, Oumar Ndour, Gabriel Ngom

**Affiliations:** 1 Service de Chirurgie Pédiatrique, Hôpital d’Enfants Albert Royer, Dakar, Sénégal; 2 Service de Chirurgie, Hôpital Régional de Ziguinchor, Université Assane Seck de Ziguinchor, Sénégal; 3 Service de Chirurgie Pédiatrique, Hôpital Aristide Le Dantec, Dakar, Sénégal; 4 Centre de Santé des HLM de Dakar, Dakar, Sénégal

**Keywords:** Pyloric atresia, newborn, rare congenital malformation

## Abstract

Pyloric atresia is a rare congenital malformation. We report a case in a 5-day newborn with pyloric atresia type C. Authors emphasize the diagnostic difficulties and therapeutic challenges in a resource-limited country.

## Introduction

Pyloric atresia (PA) is a rare congenital malformation. Accounting for less than 1% of gastro-intestinal atresia [1]. It is often isolated but may be associated with other malformations making the prognosis bad [2]. The diagnosis is suspected on the presence of non-bilious vomiting and a single gastric air bubble on abdominal X-ray. The treatment depends on the type of PA. The diagnosis of PA can be difficult and the treatment is a challenge in an area with limited resources. We report a case of pyloric atresia type C in a baby with an emphasis on diagnosis and therapeutic difficulties in an underdeveloped country.

## Patient and observation

**Figure 1:**
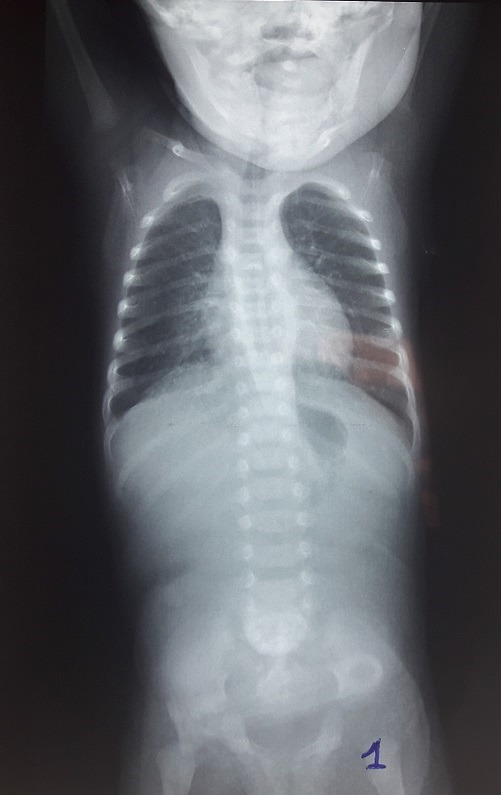
Single bubble sign

**Figure 2:**
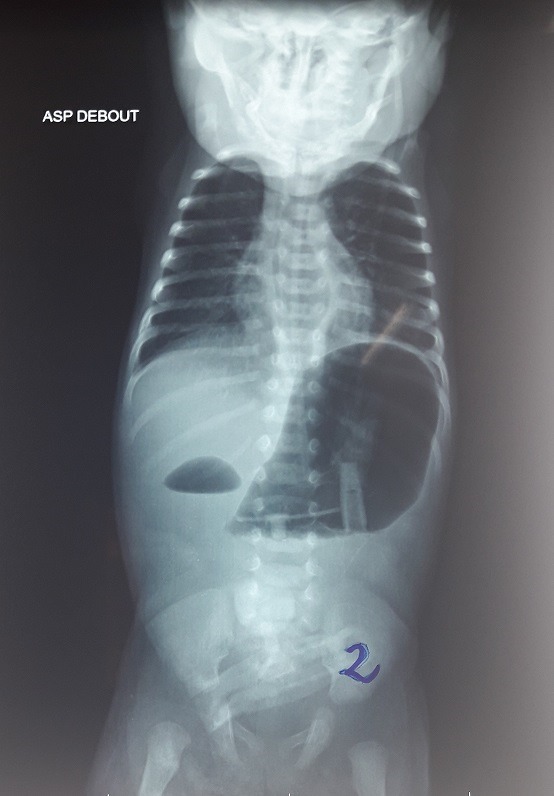
Double bubble sign

**Figure 3:**
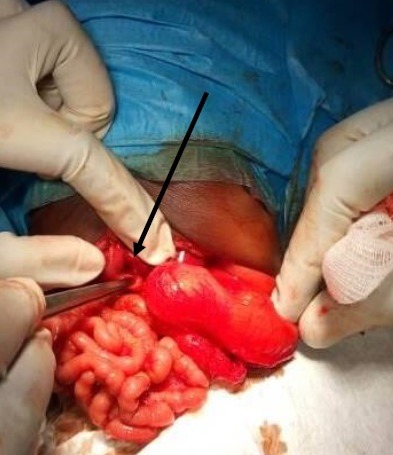
Per operatory view showing the gap between the stomach and the duodenum

A 5-day-old male weighing 1700g with a normal delivery was sent to our department for the suspicion of duodenal atresia. The newborn presented non-bilious vomiting since birth and never emitted meconium. Clinical examination found a poor general condition, a trisomic facies, a weight to 1600g and a plat abdomen. Biological check-up was normal. An abdominal X-ray showed a single bubble gastric air with no gas beyond (Figure 1). A second abdominal X-ray performed two days later showed a double bubble air without a distal aeration (Figure 2). Abdominal ultrasound was normal. The diagnosis evoked were PA and duodenal atresia. The laparotomy revelated a type C pyloric atresia with an important gap between the stomach and the duodenum (Figure 3). A duodenogastrostomy was performed and a transanastomotic naso-duodenal tube was placed. One day after surgery the newborn is fed by the nasoduodenal tube. On the 12^th^ post-operative day, this tube was removed and oral feeding is started. The baby comes out of the hospital on day 16 post-operative with a weight of 2450g. He was asymptomatic after a follow up of one year.

## Discussion

The diagnosis of PA requires a high index of suspicion because of its rarity. Despite of its specific symptoms, confusion with duodenal atresia is frequent [3]. In our observation, we evoked respectively PA and duodenal atresia based on the presence of a single bubble air and two bubbles air on abdominal X-ray. Double bubble air is described in the PA [4]. It is either linked of a pyloric membrane into the duodenum or to a reflecting configuration of the distend stomach [1, 5]. In our case this is probably the second situation, the baby having a type C of pyloric atresia. We performed duodeno-gastrostomy as recommended in type C and placed a trans anastomotic naso-duodenal tube to feed the baby very early, his general condition being bad with a low weigh before surgery. This tube is an alternative to parenteral nutrition that is not available in our hospital. It has improved the nutritional status of the baby before the beginning of oral feeding.

## Conclusion

A single gastric bubble air evokes strongly PA. However a diagnosis confusion may arise when a double bubble air appears in the same patient within a few days. For type C of PA, duodenogastrostomy with a trans-anastomotic naso-duodenal tube can be an alternative for a team that does not have parenteral nutrition because it allows to feed the baby very early and improves its general condition.

## Competing interests

The authors declare no competing interests.
